# Supplementary material for: Content-rich biological network constructed by mining PubMed abstracts
Source: BMC Bioinformatics. 2004 Oct 8;5:147. doi: 10.1186/1471-2105-5-147 (PMC528731; doi:10.1186/1471-2105-5-147)
Supplement: Additional File 2 — The original results of the above study (non-essential files are deleted to keep the file size under the limit set by BMC bioinformatics). [file 1471-2105-5-147-S2.bz2 › chilibotAdditionalFile2/dip10/279ID2498083E1102/html/FOSB_JUN.html]

 


 **FOSB** and **JUN** 
  
Found 189 abstracts in PubMed, retrieved 10.  
 

 What does Google say? 
 PDF only 
| .edu only 

---

**Interactive relationship** (e.g. stimulation, inhibition, etc)

**Neutral relationship**- Based on in vitro analyses, GnRH appeared to enhance the interaction of JunD,  **FosB** , and c Fos at the GnRHR AP 1  [ **JUN** ]  element.  Ref: 12586760 Endocrinology, 2003
- **FosB**  is a member of the AP 1  [ **JUN** ]  family of transcription factors which represent important regulators of cell proliferation and differentiation.  Ref: 12602926 Breast Cancer Res Treat, 2003
- **fosB**  was up regulated 20 fold, but only minor effects on  **jun**  variants were observed.  Ref: 12820376 Anticancer Res< MedlineTA>Anticancer Res, 2003
- Under conditions of high rhodopsin availability during exposure to light, Fra 2 and, to a minor degree,  **FosB**  substitute for c Fos and enable light induced AP 1  [ **JUN** ]  activity and thus photoreceptor apoptosis.  Ref: 12766089 Invest Ophthalmol Vis Sci, 2003
- Phosphorylated c  **Jun** , JunB, JunD, and Fra 1, but not c Fos,  **FosB** , or ATF 2, are detected in the AP 1  [ **JUN** ]  DNA binding complex in cells exposed to trivalent arsenicals.  Ref: 12475910 FASEB J, 2003
- Electrophoretic mobility shift assays using nuclear extracts of Jurkat T cells as well as TCR CD3 restimulated primary human T cells demonstrated specific binding of AP 1  [ **JUN** ] , predominantly composed of c  **Jun**  and  **FosB** , to this sequence.  Ref: 12618758 Oncogene, 2003

**Non-interactive relationship** (e.g. studied together, co-existance, homology, etc.)

- Ectopic expression of transdominant negative  **Jun**  mutants strongly reduced CD95L promoter activity and activation induced cell death, confirming the functional significance of  **FosB**  c  **Jun**  binding.  Ref: 12618758 Oncogene, 2003
- Thus, our results demonstrate an important novel function for  **FosB**  dimerized with c  **Jun**  in TCR CD3 mediated AICD in human T cells.Oncogene 2 333 1339.  Ref: 12618758 Oncogene, 2003
- Because these two proteins did not give any detectable FRET uponantigen addition, we tethered each protein with a leucine zipper motif c  **Jun**  or  **FosB**  at the C terminus to help the neighborhood of the GFP variants.  Ref: 12463362 Anal Chem, 2002
- Immunoblot analyses confirmed that c  **Jun** , JunD, JunB, c Fos, Fra 1, Fra 2 and  **FosB**  immunoreactive proteins were present in whole cell extracts of all antral follicles and midluteal phase corpora lutea as well as granulosa cells isolated from different sized antral follicles.  Ref: 12606371 Biol Reprod< MedlineTA>Biol Reprod, 2002
- Among the early response genes analyzed, c myc, junB, junD, c  **jun** , c fos,  **fosB** , fra, as well as max, mad1 4, sin3, only c  **jun**  and fra 2 mRNAs were up regulated after 5D exposure.  Ref: 12648525 J Steroid Biochem Mol Biol, 2003
- We further studied the expression of  **fosB** , c  **jun**  and junB, in the AP 1  [ **JUN** ]  complex.  Ref: 12820376 Anticancer Res< MedlineTA>Anticancer Res, 2003
- This effect was specific because palytoxin had little effect on c  **Jun** , JunB, JunD,  **FosB** , Fra 1, or Fra 2 binding or on overall levels of transcription factor binding.  Ref: 12460732 Toxicol Appl Pharmacol, 2002
- In primordial and primary unilaminar follicles, all AP 1  [ **JUN** ]  factors studied except for  **FosB**  were detected in granulosa nuclei.  Ref: 12606371 Biol Reprod< MedlineTA>Biol Reprod, 2002
